# Non-specific pain and 30-day readmission in acute coronary syndromes: findings from the TRACE-CORE prospective cohort

**DOI:** 10.1186/s12872-021-02195-z

**Published:** 2021-08-09

**Authors:** Jinying Chen, Catarina I. Kiefe, Marc Gagnier, Darleen Lessard, David McManus, Bo Wang, Thomas K. Houston

**Affiliations:** 1 Department of Population and Quantitative Health Sciences, University of Massachusetts Medical School, 368 Plantation Street, Worcester, MA 01605 USA; 2 UMass Memorial Health Care, Worcester, MA USA; 3 Department of Medicine, University of Massachusetts Medical School, Worcester, MA USA; 4 Wake Forest School of Medicine, Winston-Salem, NC USA

**Keywords:** Cardiovascular disease, Acute coronary syndrome, Non-specific pain, Readmission, Care transition, Electronic health records, Natural language processing

## Abstract

**Background:**

Patients with acute coronary syndromes often experience non-specific (generic) pain after hospital discharge. However, evidence about the association between post-discharge non-specific pain and rehospitalization remains limited.

**Methods:**

We analyzed data from the Transitions, Risks, and Actions in Coronary Events Center for Outcomes Research and Education (TRACE-CORE) prospective cohort. TRACE-CORE followed patients with acute coronary syndromes for 24 months post-discharge from the index hospitalization, collected patient-reported generic pain (using SF-36) and chest pain (using the Seattle Angina Questionnaire) and rehospitalization events. We assessed the association between generic pain and 30-day rehospitalization using multivariable logistic regression (N = 787). We also examined the associations among patient-reported pain, pain documentation identified by natural language processing (NLP) from electronic health record (EHR) notes, and the outcome.

**Results:**

Patients were 62 years old (SD = 11.4), with 5.1% Black or Hispanic individuals and 29.9% women. Within 30 days post-discharge, 87 (11.1%) patients were re-hospitalized. Patient-reported mild-to-moderate pain, without EHR documentation, was associated with 30-day rehospitalization (odds ratio [OR]: 2.03, 95% confidence interval [CI]: 1.14–3.62, reference: no pain) after adjusting for baseline characteristics; while patient-reported mild-to-moderate pain with EHR documentation (presumably addressed) was not (OR: 1.23, 95% CI: 0.52–2.90). Severe pain was also associated with 30-day rehospitalization (OR: 3.16, 95% CI: 1.32–7.54), even after further adjusting for chest pain (OR: 2.59, 95% CI: 1.06–6.35).

**Conclusions:**

Patient-reported post-discharge generic pain was positively associated with 30-day rehospitalization. Future studies should further disentangle the impact of cardiac and non-cardiac pain on rehospitalization and develop strategies to support the timely management of post-discharge pain by healthcare providers.

**Supplementary Information:**

The online version contains supplementary material available at 10.1186/s12872-021-02195-z.

## Background

Coronary heart disease affects 18.2 million adult Americans and over 1.3 million yearly hospitalizations involve acute coronary syndrome [1]. The transition from hospital to home can be challenging for patients. Patients with acute coronary syndromes often experience warning symptoms after hospital discharge, leading them back to seek acute care [2–4]. The 30-day rehospitalization rate for adult patients (≥ 18 year of age) has ranged from 10 to 17% [1, 5, 6].

Evaluation of patients’ post-discharge symptoms is an integral part of transitional care to improve clinical outcomes including readmission [7]. Pain (both cardiac and non-cardiac) is common in post-discharge patients with acute coronary syndromes [8–13]. Research evidence of the impact of pain on healthcare utilization is valuable to transitional care, but was mainly on cardiac pain for this patient population [2, 3, 8–10]. Little is known about generic pain and its association with rehospitalization.

In this study, we evaluated patient-reported generic pain within 30-days post-discharge and its association with 30-day rehospitalization in patients with acute coronary syndromes, using data from a prospective cohort study, the Transitions, Risks, and Actions in Coronary Events Center for Outcomes Research and Education (TRACE-CORE) project [14]. We studied generic, not just cardiac, pain because in a previous study we found that generic pain was associated with 30-day acute care use in patients with heart failure [15]. Also, although cardiac pain is typical for patients with acute coronary syndromes, other types of pain (e.g., non-cardiac chest pain, wound pain from surgery, and chronic pains) are also common [11–13, 16–18]. Recent studies found that non-cardiac or non-specific chest pain was prevalent in readmissions in patients with acute coronary syndromes [11, 12, 19, 20]. Further, the cardiac nature of pain is not always apparent, as is captured in the nomenclature “atypical chest pain” and “non-specific chest pain” [21, 22], which may refer to generic pain or other angina-equivalent symptoms. We hypothesized that post-discharge patient-reported generic pain would be associated with 30-day rehospitalization. In addition, we examined the associations among patient-reported pain, documentation of pain in electronic health record (EHR) notes, and rehospitalization. Mentions of pain in EHR notes were automatically identified by natural language processing (NLP).

## Methods

### Data sources

Data for this study were obtained from the TRACE-CORE cohort study [14] and enhanced by linking the original TRACE-CORE data to patients’ electronic health record notes within the healthcare system of their index hospitalization (UMass Memorial Health Care—UMMHC in Worcester, MA).

### Study design

In TRACE-CORE, patients hospitalized for acute coronary syndromes were followed prospectively through patient interviews at 1, 3, 6, 12 months post-discharge and medical record abstraction for 24 months [14]. We examined pain reported by TRACE-CORE patients at one month post-discharge and its association with 30-day rehospitalization.

### Setting and sample

The details of the study setting and patient recruitment have been documented for TRACE-CORE elsewhere [14, 23]. Briefly, patients were recruited from 6 study sites (hospitals), which serve the majority of patients hospitalized with acute coronary syndromes in central Massachusetts and central Georgia, between April 2011 and May 2013 [14, 23]. The cohort included adult (over 21 years old) patients who had at least one of: (1) serial ECG changes consistent with ischemia; (2) elevation of cardiac biomarkers; (3) cardiac catheterization revealing over 70% stenosis in a coronary artery; and (4) admission for urgent or rescue PCI/CABG, with symptoms of acute ischemia in the 72 h prior to admission. Exclusion criteria included pregnancy, prison custody, receiving palliative care, dementia, acute coronary syndrome secondary to demand ischemia or aortic dissection, perioperative acute coronary syndrome, admission for trauma or elective cardiac catheterization procedure, and transfer from another hospital after staying at the referring hospital for over 24 h.

For this study, we included the TRACE-CORE patients who were discharged from two hospitals that comprise UMMHC and also completed the assessment of generic pain at on-month follow-up (N = 787). We chose this dataset because we had access to EHR notes from UMMHC for the TRACE-CORE patients for additional analyses involving EHR-documented pain.

Institutional Review Board (IRB) approval was obtained for the parent TRACE-CORE study from the Committee for the Protection of Human Subjects in Research at the University of Massachusetts Medical School (UMMS) and the other study sites. All participants provided written informed consent. Additional IRB approval was obtained from the Committee for the Protection of Human Subjects in Research at UMMS to access EHR notes for this study.

### Data collection

#### Baseline variables

Baseline data for each patient were collected through a computer-assisted 60-min structured interview during patient’s index hospitalization or by phone call within 72 h post-discharge, which included patient’s demographics, socioeconomic factors, healthcare seeking behavior, and other background information. Patient’s medical history, comorbidities, and cardiac procedures were obtained through medical record abstraction. Healthcare seeking behavior was assessed by two questions “Is there a place that you usually go to when you are sick or need advice about your health?” (with 3 response options: (1) yes, (2) there is no place, and (3) there is more than one place) and “What kind of place is it—a clinic, doctor’s office, emergency room, or some other place?” (with 4 response options (1) clinic, (2) doctor’s office, (3) hospital emergency room, and (4) some other place”). Because only patients who answered (1) “yes” to question 1 have responded to question 2 in our data, we combined responses to these two questions into a single 4-category variable (see Table 1). We combined response options (1) “clinic” and (2) “doctor’s office” for question 2 into one category that represented the non-acute care setting. This treatment was informed by prior studies in health services research that separated non-acute care and acute care when studying factors impacting hospital admissions and strategies to reduce admissions [24–26]. Comorbidity was estimated by counting diseases from 14 medical conditions (see footnote in Table 1) documented in patient’s medical history. Table 1 names baseline variables included in this analysis.

**Table 1:** Patient characteristics at baseline, by pain self-reported one month after discharge: TRACE-CORE, 2011–2013

Variable	All Patients	Categories of self-reported pain after discharge				
		No pain	Mild-moderate pain, documented in EHR	Mild-moderate pain, NOT documented in EHR	Severe pain	*P* value
	N = 787	N = 285	N = 101	N = 347	N = 54	
	N (%)	N (%)	N (%)	N (%)	N (%)	
*Age*						0.01*
< 50	116 (14.7)	30 (10.5)	14 (13.9)	64 (18.4)	8 (14.8)	
50–64	336 (42.7)	120 (42.1)	47 (46.5)	141 (40.6)	28 (51.9)	
≥ 65	335 (42.6)	135 (47.4)	40 (39.6)	142 (40.9)	18 (33.3)	
*Gender*						0.01*
Female	235 (29.9)	68 (23.9)	26 (25.7)	121 (34.9)	30 (37.0)	
*Race*						0.42
White	741 (94.9)	269 (95.1)	97 (97.0)	326 (94.8)	49 (90.7)	
Black	8 (1.0)	4 (1.4)	1 (1.0)	3 (0.9)	0 (0.0)	
Hispanic	32 (4.1)	10 (3.5)	2 (2.0)	15 (4.4)	5 (9.3)	
*Education*						0.31
≤ High school	286 (36.3)	115 (40.4)	32 (31.7)	116 (33.4)	23 (42.6)	
Some college	241 (30.6)	75 (26.3)	33 (32.7)	116 (33.4)	17 (31.5)	
≥ College graduate	260 (33.0)	95 (33.3)	36 (35.6)	115 (33.1)	14 (25.9)	
*Low social support*						0.38
Yes	41 (5.2)	17 (6.0)	5 (5.0)	14 (4.0)	5 (9.3)	
*Live alone*						0.63
Yes	158 (20.1)	54 (18.9)	24 (23.8)	67 (19.3)	13 (24.1)	
*Low health literacy*						0.69
Yes	227 (29.0)	80 (28.2)	34 (34.0)	97 (28.0)	16 (29.6)	
*Heavy drinker*						0.30
Yes	74 (9.4)	25 (8.8)	13 (12.9)	34 (9.8)	2 (3.7)	
*Current smoker*						0.04*
Yes	161 (20.6)	52 (18.2)	23 (22.8)	68 (19.6)	19 (35.2)	
*Where to seek health care*						0.44
No places	87 (11.1)	28 (10.0)	11 (10.9)	42 (12.1)	6 (11.1)	
Clinic or doctor office	619 (79.2)	233 (82.9)	78 (77.2)	267 (77.2)	41 (75.9)	
Other places	35 (4.5)	8 (2.8)	4 (4.0)	21 (6.1)	2 (3.7)	
Emergency department	41 (5.2)	12 (4.3)	8 (7.9)	16 (4.6)	5 (9.3)	
*Acute coronary syndrome type*						0.01*
STEMI	171 (21.7)	72 (25.3)	10 (9.9)	79 (22.8)	10 (18.5)	
NSTEMI	470 (59.7)	174 (61.1)	65 (64.4)	200 (57.6)	31 (57.4)	
Unstable angina	146 (18.6)	39 (13.7)	26 (25.7)	68 (19.6)	13 (24.1)	
*History of PCI*						0.12
Yes	174 (22.1)	54 (18.9)	22 (21.8)	80 (23.1)	18 (33.3)	
*History of CABG*						0.33
Yes	87 (11.1)	26 (9.1)	16 (15.8)	39 (11.2)	6 (11.1)	
Comorbidity^a^ (mean [SD])	2.0 [1.7]	1.8 [1.6]	2.3 [1.8]	2.0 [1.7]	2.8 [2.0]	< 0.001*
*Reporting generic pain*						< 0.001*
No	181 (23.1)	107 (37.8)	11 (10.9)	58 (16.8)	5 (9.3)	
Mild to moderate	447 (57.1)	141 (49.8)	65 (64.4)	220 (63.8)	21 (38.9)	
Severe	155 (19.8)	35 (12.4)	25 (24.8)	67 (19.4)	28 (51.9)	

Pain category was defined using pain self-reported for the first month after discharge from index hospitalization (using SF-36 survey distributed at one month post-discharge) and electronic health record (EHR) documentation of pain extracted by natural language processing from patient’s clinical notes created within 30 days post discharge and before readmission to the hospital (if any). *TRACE-CORE* Transitions, Risks, and Actions in Coronary Events Center for Outcomes Research and Education, *STEMI* ST-segment elevation myocardial infarction, *NSTEMI* non-ST segment elevation myocardial infarction, *PCI* percutaneous coronary intervention, *CABG* coronary artery bypass graft, *SD*, standard deviation

^a^The number of diseases from the following 14 conditions in patient’s medical history: atrial fibrillation, Alzheimer or dementia, anemia, cancer, congestive heart failure, chronic kidney disease, chronic lung disease, Type 2 diabetes, dialysis, hypertension, peripheral vascular disease, valvular heart disease, coronary heart disease or myocardial infarction, transient ischemic attack or stroke

*Indicates statistically significant (*P* < 0.05). *p*-values were calculated by chi-square test (for non-age categorical variables that have no cell size smaller than 5), Fisher’s exact test (for non-age categorical variables that have cell size smaller than 5), analysis of variance (for comorbidity). We used Cuzick's Test for Trend to assess the trend of age across the 4 pain categories

#### Patient-reported post-discharge pain status

Patient’s post-discharge health-related quality of life, including pain, was assessed by the structured phone interview at one month post-discharge. The generic pain status was assessed by “How much bodily pain have you had during the past 4 weeks?—none, very mild, mild, moderate, severe, very severe” through SF-36 [27]. Based on sample size, we grouped responses with “very mild”, “mild”, and “moderate” into a single category mild-to-moderate and grouped “severe” and “very severe” into severe in our analysis. Chest pain was assessed by the Seattle Angina Questionnaire (SAQ), “Over the past 4 weeks, on average, how often have you had chest pain, chest tightness, or angina?—4 or more times per day, 1–3 times per day, 3 or more times per week but not every day, 1–2 times per week, less than once a week, None over the past 4 weeks” [28]. We grouped the first five categories into a single category having-chest-pain in our analysis.

#### Pain status documented in post-discharge EHR notes

We used NLP to categorize whether patients had documentation of pain in clinical notes. NLP has been increasingly used to process EHR data, including extracting symptoms from clinical notes [29]. Researchers have also used NLP-identified symptoms for health services research [30–34].

For this study, we developed and validated an NLP system to identify pain symptoms from EHR notes [35]. In brief, using a knowledge-driven method, we adapted a general-purpose clinical NLP system cTAKES [36] to pain extraction. cTAKES extracts and maps medical terms to Unified Medical Language System concepts [36]. To adapt cTAKES to the pain domain, we first compiled a list of terms related to pain (e.g., pain, tenderness, headache, angina, etc.) using input from domain experts and then converted the term list to regular expressions to match cTAKES-recognized terms. We evaluated the system using 200 in-patient and out-patient notes (11,917 sentences), with each sentence labeled by a physician as mentioning pain or not. An EHR note was labeled as documenting pain if it contained at least one sentence that mentioned pain. We measured the system’s performance by three metrics commonly used for evaluating NLP: precision, recall, and the F1 score. In our case, precision is the number of pain instances correctly identified by NLP divided by the number of pain instances identified by NLP; recall is the number of pain instances correctly identified by NLP divided by the number of pain instances identified or labeled by domain experts; F1 is the harmonic mean of precision and recall. A pain instance is a sentence (for sentence-level evaluation) or an EHR note (for note-level evaluation) that documented pain. The system achieved 0.80 precision, 0.91 recall, and 0.85 F1 for pain extraction at the sentence level and 0.89 precision, 0.98 recall, and 0.93 F1 at the EHR note level.

We applied the system to clinical notes created after a patient’s discharge from index hospitalization and before rehospitalization (if any) to identify pain symptoms. If a patient was not re-hospitalized before the 1-month follow-up, we sought pain documentation in the EHR for the entire 1-month period. When applying NLP, we used a rule-based method to identify and exclude the “History of Present Illness” sections, which often documented patients’ chest pain related to their index hospitalizations rather than pain occurred after hospital discharge. EHR notes created after a patient’s rehospitalization could also document pain. We excluded those notes from our analysis, because we wanted to use the NLP-identified EHR-documented pain as a conceptual proxy for whether there was any provider-patient engagement around the patient’s pain status before readmission (if any) during the 30-day post-discharge period, i.e., whether the pain was “addressed”.

#### Outcome variable

Patients’ self-reported rehospitalizations within 30 days post-discharge were confirmed by chart review in the study hospitals when possible.

### Data analysis

We first compared baseline patient characteristics across four pain categories defined by patient-self-reported pain at 1-month follow-up and EHR-documented pain using chi-square test, Fisher’s exact test, Cuzick's Test for Trend [37], or analysis of variance as appropriate. The four pain categories were: (1) self-reporting no pain; (2) self-reporting mild-to-moderate pain and having EHR-documented pain before readmission (if any); (3) reporting mild-to-moderate pain but having no EHR-documented pain before readmission (if any); and (4) reporting severe pain. We did not separate patients reporting severe pain base on whether they had EHR-documented pain because only 2 patients reported severe pain, had EHR-documented pain, and were readmitted. This sample size is too small for the secondary analysis described below.

We then reported descriptive statistics about patient-self-reported pain and NLP-identified EHR documentation of pain. In a post-study analysis, we reviewed cases where patients did not report generic pain in survey but had NLP-identified EHR documentation of pain, and categorized the reasons for mismatch.

In the primary analysis, we first assessed the incidence of the outcome (i.e., 30-day rehospitalization) across the levels of patient-reported generic pain status (not considering EHR documentation) and the unadjusted association between patient’s pain status and the outcome. We then used multivariable logistic regression to adjust for potential confounding by (1) patient characteristics collected in index hospitalization and (2) patient characteristics collected in index hospitalization plus patient-reported chest pain at 1-month follow-up. We identified potential confounders from statistical analysis (Table 1, *P* < 0.05). We also adjusted for healthcare seeking behaviors for both models 2 and 3.

Further, we conducted a secondary analysis to assess the relationship between the outcome, patient-self-reported pain, and EHR documentation of pain. We used the variable that represents the four pain categories (defined at the beginning of this section) as the predictor variable, adjusting for covariates as same as defined for the primary analysis.

As a sensitivity analysis to further disentangle the effects of chest pain versus generic pain, we repeated the association analyses, limiting them to patients not self-reporting chest pain by SAQ (n = 530).

Statistical significance was defined as a two-tailed *p*-value of < 0.05. Statistical analyses were programmed using STATA/IC 15.1 (StataCorp LLC, Texas, USA) [38].

## Results

### Patient characteristics

Among the 787 study patients, mean age was 62.0 (SD = 11.4), 29.9% were women, and 5.1% were Black or Hispanic individuals (Table 1). Age, sex, smoking status, type of acute coronary syndrome, comorbidity, and patient-self-reported pain at index hospitalization were significantly different across the 4 pain categories. Compared with all TRACE-CORE participants (N = 2174), the patients analyzed in our study (N = 787) had similar mean age (62 vs. 61), less females (29.9% vs. 33.5%), more non-Hispanic white (94.9% vs. 81.0%), and better socioeconomic status (see Additional file 1 for details). The difference was expected, because the parent TRACE-CORE study recruited patients from 6 hospitals in 3 cities of 2 states in U.S. These participating study sites served a heterogeneous patient population and were selected purposely for their sociodemographic and socioeconomic diversity [14, 39].

### Self-reported post-discharge generic pain and chest pain

Among the 787 patients, 285 (36.2%) reported no pain, 448 (56.9%) reported mild-to-moderate pain, and 54 (6.9%) reported severe pain; 770 (97.8%) responded to the SAQ chest pain question at 1-month follow-up, with 530 (68.8%) reporting no chest pain.

### EHR documentation of pain

NLP identified EHR documentation of pain for 150 (19.1%) patients. While the likelihood of finding pain in the EHR by NLP increased with pain severity (trend test *P* < 0.001, Table 2), only 22.5% of patients reporting mild-to-moderate pain and 27.8% of patients reporting severe pain had NLP-identified EHR-documented pain. 34 (11.9%) patients not self-reporting pain were identified by NLP as having pain. Among these 34 patients, NLP was wrong for 9 (26%) cases, most of which were negations of pain (see Additional file 2).

**Table 2:** Patient-self-reported pain and EHR documentation of pain

	Patient self-reported pain			*P* value
	No pain	Mild-to-moderate pain	Severe pain	
	N = 285	N = 448	N = 54	
*EHR documentation of pain, N (%)*				< 0.001*
No	251 (88.1)	347 (77.5)	39 (72.2)	
Yes	34 (11.9)	101 (22.5)	15 (27.8)	

Patient-self-reported pain status was collected for the first month after discharge from index hospitalization (using SF-36 survey distributed at one month post-discharge). Electronic health record (EHR) documentation of pain was extracted by natural language processing from patient’s clinical notes created within 30 days post discharge and before readmission to the hospital (if any)

*Indicates statistically significant (*P* < 0.05). We used Cuzick's Test for Trend to assess the trend of EHR-documentation of pain across the severity levels of patient self-reported pain

### Patient-reported pain and 30-day rehospitalization

Within 30 days post-discharge, 87 (11.1%) patients were hospitalized. There was a trend of increased rehospitalization rate from no pain, mild-to-moderate pain, and severe pain (7.4% vs. 12.3% vs. 20.4%, Table 3, Column 1). Compared to patients reporting no pain, those reporting mild-to-moderate (odds ratio [OR]: 1.76, 95% confidence interval [CI]: 1.04–2.98) or severe (OR: 3.22, 95% CI: 1.45–7.14) pain had a significantly higher risk of rehospitalization (Model 1). This trend remained after adjusting for factors from index hospitalization (Model 2). After further adjusting for patient-reported chest pain, severe pain again had a higher rehospitalization rate than no pain (OR: 2.59, 95% CI: 1.06–6.35; Model 3), but mild-to-moderate pain was no longer significant.

**Table 3:** Association of self-reported post-discharge pain status with 30-day rehospitalization: TRACE-CORE, 2011–2013

Pain condition	Incidence of 30-day rehospitalization	Model 1^a^: Unadjusted		Model 2^b^: Adjusted for baseline characteristics		Model 3^c^: Model 2 further adjusted for self-reported chest pain	
	n/N (%)	OR (95% CI)	*P* value	OR (95% CI)	*P* value	OR (95% CI)	*P* value
No pain	21/285 (7.4)	Reference		Reference		Reference	
Mild to moderate pain	55/448 (12.3)	1.76 (1.04–2.98)	0.04	1.84 (1.05–3.24)	0.03*	1.43 (0.78–2.60)	0.25
Severe pain	11/54 (20.4)	3.22 (1.45–7.14)	< 0.01*	3.16 (1.32–7.54)	0.01*	2.59 (1.06–6.35)	0.04*

Patient-self-reported pain status was collected for the first month after discharge from index hospitalization (using SF-36 survey distributed at one month post-discharge)

*TRACE-CORE* Transitions, Risks, and Actions in Coronary Events Center for Outcomes Research and Education

^a^Model 1: logistic regression, unadjusted for covariates

^b^Model 2: multivariable logistic regression, adjusted for age, sex, smoking status, acute coronary syndrome type, comorbidity, patient-self-reported generic pain, and healthcare seeking behaviors reported at index hospitalization

^c^Model 3: Model 2 further adjusted for patient-report of chest pain, tightness, or angina on the Seattle Angina Questionnaire

*Indicates statistically significant (*P* < 0.05)

### Patient-reported pain (accounting for EHR documentation) and 30-day rehospitalization

**Fig. 1:**
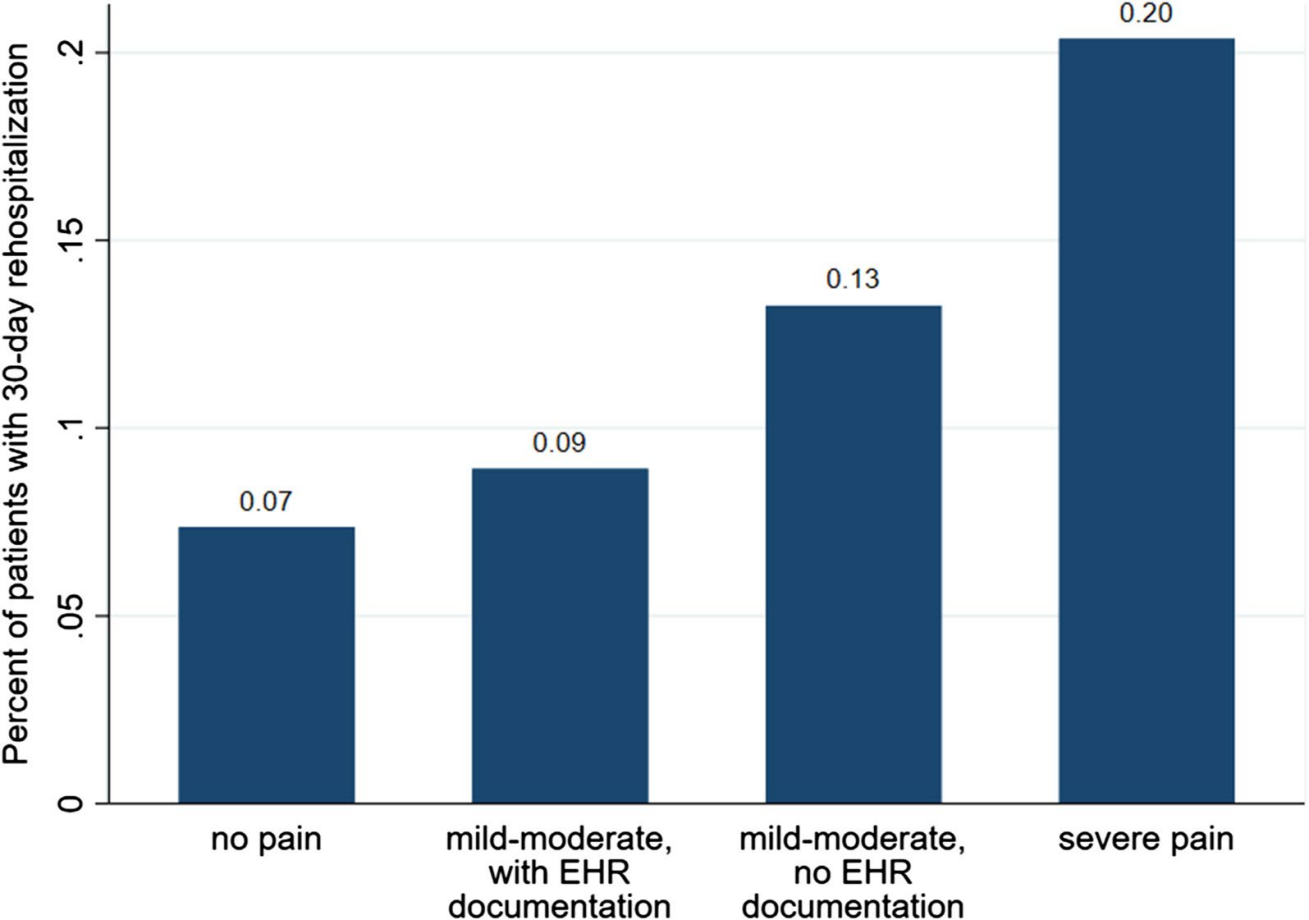
Prevalence of 30-day rehospitalization in patients across the 4 pain categories (trend test *P* = 0.002*). Patient-self-reported pain status was collected for the first month after discharge from index hospitalization (using SF-36 survey distributed at one month post-discharge). Electronic health record (EHR) documentation of pain was identified by natural language processing from clinical notes documented within 30 days post-discharge and before readmission (if any). *Indicates statistically significant (*P* < 0.05). We used Cuzick's Test for Trend to assess the trend of increased 30-day rehospitalization rates across the 4 pain categories

As shown in Table 4 and Fig. 1, the rehospitalization rate increased across the four pain conditions (7.4% vs. 8.9% vs. 13.3% vs. 20.4%, trend test *P* = 0.002). Patients reporting mild-to-moderate pain but having no EHR-documented pain before readmission had a higher rehospitalization rate than those reporting no pain (OR: 1.92, 95% CI: 1.12–3.30; Model 1). However, for those who reported mild-to-moderate pain and did have EHR-documented pain, their risk of rehospitalization was not different from those reporting no pain. After full adjustment (Model 3), those with mild-to-moderate pain but no EHR-documented pain still had a relative higher risk (OR: 1.56, 95% CI: 0.84–2.90), although not significantly different. Among the 54 patients who self-reported severe pain, 15 had EHR-documentation of pain and 2 (13.3%) of them were readmitted; 39 had no EHR-documentation of pain and 9 (23.1%) of them were readmitted.

**Table 4:** Association of pain category (combining self-report with EHR data) with 30-day rehospitalization: TRACE-CORE, 2011–2013

Pain condition	Incidence of 30-day rehospitalization	Model 1^a^: Unadjusted		Model 2^b^: Adjusted for baseline characteristics		Model 3^c^: Model 2 further adjusted for self-reported chest pain	
	n/N (%)	OR (95% CI)	*P* value	OR (95% CI)	*P* value	OR (95% CI)	*P* value
No pain	21/285 (7.4)	Reference		Reference		Reference	
Mild to moderate pain, documented in EHR	9/101 (8.9)	1.23 (0.54–2.78)	0.62	1.23 (0.52–2.90)	0.63	0.99 (0.42–2.40)	1.00
Mild to moderate pain, not documented in EHR	46/347 (13.3)	1.92 (1.12–3.30)	0.02*	2.03 (1.14–3.62)	0.02*	1.56 (0.84–2.90)	0.16
Severe pain	11/54 (20.4)	3.22 (1.45–7.14)	0.004*	3.11 (1.30–7.42)	0.01*	2.57 (1.05–6.29)	0.04*

Patient-self-reported pain status was collected for the first month after discharge from index hospitalization (using SF-36 survey distributed at one month post-discharge). Electronic health record (EHR) documentation of pain was extracted by natural language processing from patient’s clinical notes created within 30 days post discharge and before readmission to the hospital (if any)

TRACE-CORE indicates Transitions, Risks, and Actions in Coronary Events Center for Outcomes Research and Education

^a^Model 1: logistic regression, unadjusted for covariates

^b^Model 2: multivariable logistic regression, adjusted for age, sex, smoking status, acute coronary syndrome type, comorbidity, patient-self-reported generic pain, and healthcare seeking behaviors reported at index hospitalization

^c^Model 3: Model 2 further adjusted for patient-report of chest pain, tightness, or angina on the Seattle Angina Questionnaire

*Indicates statistically significant (*P* < 0.05)

### Sensitivity analysis

Of the 787 patients, 530 reported no chest pain during the past four weeks by SAQ at one-month follow-up. When we repeated all analyses restricted to this smaller sample, the findings were qualitatively similar, with point estimates for prevalence and odds ratios all in the same direction as in our main analyses, but no longer statistically significant (see Additional file 3).

## Discussion

We found that after discharge from a hospitalization for acute coronary syndrome, patient-reported generic pain was prevalent and was associated with rehospitalization. After adjusting for patient-reported chest pain and other covariates, severe generic pain was still associated with rehospitalization. Going beyond our prior work that studied the association of pain and readmission [15], by using the TRACE-CORE data and NLP, we were able to explore patient-provider engagement around pain, i.e., documentation of pain (presumably addressed) identified by NLP from clinical notes, within 30 days post-discharge. Notably, when pain was both self-reported and documented in the EHR, the rehospitalization rate was not significantly higher than that for patients reporting no pain (8.9% vs. 7.4%, *P* = 0.62). However, the rehospitalization rate when pain was self-reported but NOT documented in the EHR was significantly higher than no pain (13.3% vs. 7.4%, *P* = 0.02). Below, we further explore our findings by highlighting the unique aspects and discuss their implications.

We found a “dose–response” increase of 30-day rehospitalization rates across post-discharge pain severity levels, from no pain to mild/moderate pain to severe pain (Table 3), which persisted after adjusting for clinical factors collected from the index hospitalization. These results were compatible with our previous finding that patient-reported post-discharge generic pain was associated with increased risk of 30-day acute care use [15]. Our prior results were in heart failure patients who reported post-discharge pain through daily automated telephone assessments [40]. Taken together, both studies suggest that monitoring patient’s pain status after discharge may be valuable in identifying high-risk patients for early intervention to improve patient outcomes including readmission. However, prior studies do not address the questions: What happens if the patient’s pain is addressed by a healthcare provider? How will this impact readmission?

Using NLP methods, we examined presumed patient-provider engagement around pain before patient’s rehospitalization and assessed its impact on rehospitalization (Table 4). NLP revealed that, among patients self-reporting post-discharge pain, less than 20% had documentation of pain in their clinical notes before readmission, suggesting a lack of early attention to patients’ pain symptoms by providers. Even after adjusting for multiple covariates from the index hospitalization, patients with mild-to-moderate pain NOT documented in the EHR had more than twice the odds of readmission than no pain (OR: 2.03, 95% CI: 1.14–3.62). In contrast, the odds ratio for mild-to-moderate pain documented in the EHR versus no pain was not significantly different from one (OR: 1.23, 95% CI: 0.52–2.90). Thus, our findings are consistent with a clinical scenario where, for patients with pain, having pain attended by healthcare providers improves patients’ ability to stay at home to reduce early readmissions.

It is worth noting that our study used patient-self-reported pain collected by the 1-month follow-up survey (SF-36), which queried patient’s pain status during the past four weeks. We therefore cannot completely confirm that patient-reported pain always began before readmission. It is possible that patient-reports of pain began before readmission. This is compatible with our analysis results and the scenario discussed in the previous paragraph (Table 4). In addition, patients who reported severe pain had more EHR-documentation of pain before readmission compared with patients who reported mild-to-moderate pain (Table 2). This is consistent with a scenario where patients who had severe pain were more likely to seek care before readmission than those with mild-to-moderate pain. If the majority of patient-reports of pain began after readmission, it would be less likely to see these patterns from our data. To be sure, it is also possible that some patients were first readmitted to the hospital and then had new pain. For these patients, the new pain could be related to treatment received during rehospitalization or to other diseases. Future studies that include daily assessments of pain will help us better understand the directionality of the association between pain and readmission.

In studying post-discharge care and patient reports of pain, researchers and clinicians should take care to distinguish between cardiac and non-cardiac pain, especially in patients with acute coronary syndromes. In this study, we tried to separate the effects of cardiac and non-cardiac pains on readmission by adjusting for patient-reported chest pain through the SAQ. After adjusting for this factor (in addition to other covariates), the association between severe pain and 30-day rehospitalization remained significant; while mild-to-moderate pain (Model 3 in Table 3) and unaddressed mild-to-moderate pain (Model 3 in Table 4) were not significant any more. Thus, patient-reported chest pain is a partial confounder of the association between generic pain and readmission. Our sensitivity analyses, using only patients without self-reported chest pain—a much smaller sample, provided point estimates similar to the main analyses, although not statistically significant. This suggest that generic pain may, indeed, be an independent factor associated with rehospitalization. Note that separating effects of cardiac and non-cardiac pain is challenging in the real-world settings. First, multimorbidity is prevalent in older patients with cardiovascular disease [41]. In our sample, patient-reported chest pain (SAQ) and generic pain (SF-36) co-occur frequently. Second, there is a measurement challenge. SAQ asks patients to report chest pain but cannot fully distinguish between cardiac and non-cardiac pain. Non-cardiac chest pain is common in patients with acute coronary syndromes [11, 12], and it is difficult for patients to distinguish between cardiac and non-cardiac chest pain [42, 43]. It is possible that patients would report both cardiac and non-cardiac pain (e.g., chest pains with musculoskeletal, gastrointestinal, or respiratory origin [22, 44, 45]) when responding to SAQ. From the analytic perspective, more patients with generic pain and no reports of cardiac pain would be helpful in isolating the association of interest. From the perspective of patient care, the measurement challenge and the literature documenting anxiety, fear, physical disability, and high healthcare utilization in patients with non-cardiac chest pain [43, 46–48] suggest that early evaluation and treatment of post-discharge chest pain may enhance patient safety and quality of life and also reduce excessive healthcare cost.

By using NLP, we were able to quantify EHR documentation of pain for the large sample of patients in this study. However, like other artificial intelligence technologies, NLP can make mistakes. As a means of quality control, we validated our NLP system on 200 clinical notes, which showed decent results including a high recall (see Data Collection), before applying it to this study. Our post-study analysis suggests that the NLP system needs further improvement in detecting negation, which we will address in future.

Our study has clear limitations. We used observational data, which, of course, precludes definitive causal inferences. Also, our data lack detailed information on the nature of post-discharge pian, such as onset, location, and duration. The 1-month follow-up survey queried patient’s pain status during the past four weeks, we therefore cannot ascertain every patient-reported pain occurred before readmission. Importantly, we used EHR documentation of pain as a proxy for the pain having been addressed by a clinician, which may not have occurred; and the lack of EHR documentation of pain may mean lack of care overall. To partially address this last possibility, we used patient-self-reported healthcare seeking behavior to adjust the analysis. We hope that our findings from this exploratory study will serve as a catalyst for future in-depth research about post-discharge non-specific pain in patients with acute coronary syndromes.

## Conclusions

We found that patient-reported post-discharge generic pain was positively associated with 30-day rehospitalization in acute coronary syndromes. Our results are also consistent with a clinical scenario where provider’s early attention to patients’ pain could possibly reduce readmissions. Although we could not completely separate the effects of cardiac and non-cardiac pain, our results suggest that the effect of non-cardiac pain on readmission deserves further study. Non-cardiac chest pain has been associated with high resource utilization and cost [43, 46] and is common in readmissions in patients with acute coronary syndromes [11, 12]. Early evaluation of patient-reported chest pain may improve risk stratification to optimize the use of healthcare resources. Further, early treatment of non-chest pain can help prevent deterioration of other diseases that may cause readmission and also improve patient’s quality of life. Although the billable Transitional Care Management services supported by the Centers for Medicare and Medicaid Services has included symptom assessment in its follow-up telephone call [49], it is unlikely that a single, provider-initiated follow-up call will capture all warning symptoms at the right time, or at all. Intensive transitional care programs [50–52] that offer multiple follow-ups are expensive and difficult to scale up. It is thus important and beneficial to develop technology-based strategies (e.g., symptom tracking tools) to support timely patient-provider communication about warning symptoms to improve the processes and outcomes of transitional care.

## Supplementary Information

**Additional file 1.** Comparison of characteristics between patients analyzed in this study and all TRACE-CORE participants.

**Additional file 2.** Example cases where NLP identified pain not reported by the SF-36 survey.

**Additional file 3.** Sensitivity analyses, using patients not self-reporting chest pain (N = 530).

## Data Availability

The datasets generated and analyzed during the study are not publicly available because they contain protected health information (PHI) but de-identified data are available from the corresponding author on reasonable request.

## Abbreviations

EHR: Electronic health record
IRB: Institutional Review Board
NLP: Natural language processing
SAQ: Seattle Angina Questionnaire
TRACE-CORE: Transitions, Risks, and Actions in Coronary Events Center for Outcomes Research and Education
UMMHC: UMass Memorial Health Care
UMMS: University of Massachusetts Medical School

## References

[1] Benjamin EJ, Muntner P, Bittencourt MS (2019). Heart disease and stroke statistics-2019 update: a report from the American Heart Association. Circulation.

[2] Southern DA, Ngo J, Martin BJ, Galbraith PD, Knudtson ML, Ghali WA (2014). Characterizing types of readmission after acute coronary syndrome hospitalization: implications for quality reporting. J Am Heart Assoc.

[3] Dharmarajan K, Hsieh AF, Lin Z, Bueno H, Ross JS, Horwitz LI (2013). Diagnoses and timing of 30-day readmissions after hospitalization for heart failure, acute myocardial infarction, or pneumonia. JAMA.

[4] Shah M, Patil S, Patel B, Agarwal M, Davila CD, Garg L (2018). Causes and predictors of 30-day readmission in patients with acute myocardial infarction and cardiogenic shock. Circ Heart Fail.

[5] Khera R, Jain S, Pandey A, Agusala V, Kumbhani DJ, Das SR (2017). Comparison of readmission rates after acute myocardial infarction in 3 patient age groups (18 to 44, 45 to 64, and ≥ 65 years) in the United States. Am J Cardiol.

[6] Kim LK, Yeo I, Cheung JW, Swaminathan RV, Wong SC, Charitakis K (2018). Thirty-Day readmission rates, timing, causes, and costs after ST-Segment–Elevation myocardial infarction in the United States: a national readmission database analysis 2010–2014. J Am Heart Assoc.

[7] Enderlin CA, McLeskey N, Rooker JL, Steinhauser C, D'Avolio D, Gusewelle R (2013). Review of current conceptual models and frameworks to guide transitions of care in older adults. Geriatr Nurs.

[8] Beinart SC, Sales AE, Spertus JA, Plomondon ME, Every NR, Rumsfeld JS (2003). Impact of angina burden and other factors on treatment satisfaction after acute coronary syndromes. Am Heart J.

[9] Arnold SV, Morrow DA, Lei Y, Cohen DJ, Mahoney EM, Braunwald E (2009). Economic impact of angina after an acute coronary syndrome: insights from the MERLIN-TIMI 36 trial. Circ Cardiovasc Qual Outcomes.

[10] Wasfy JH, Strom JB, O’Brien C, Zai AH, Luttrell J, Kennedy KF (2014). Causes of short-term readmission after percutaneous coronary intervention. Circ Cardiovasc Interv.

[11] Kwok CS, Wong CW, Shufflebotham H, Brindley L, Fatima T, Shufflebotham A (2017). Early readmissions after acute myocardial infarction. Am J Cardiol.

[12] Qintar M, Spertus JA, Tang Y, Buchanan DM, Chan PS, Amin AP (2017). Noncardiac chest pain after acute myocardial infarction: frequency and association with health status outcomes. Am Heart J.

[13] Lahtinen P, Kokki H, Hynynen M (2006). Pain after cardiac surgery—a prospective cohort study of 1-year incidence and intensity. Anesthesiol: J Am Soc Anesthesiol.

[14] Waring ME, McManus RH, Saczynski JS, Anatchkova MD, McManus DD, Devereaux RS (2012). Transitions, Risks, and Actions in Coronary Events-Center for Outcomes Research and Education (TRACE-CORE): design and rationale. Circ Cardiovasc Qual Outcomes.

[15] Chen J, Sadasivam R, Blok AC, Ritchie CS, Nagawa C, Orvek E (2020). The association between patient-reported clinical factors and 30-day acute care utilization in chronic heart failure. Med Care.

[16] Zubrzycki M, Liebold A, Skrabal C, Reinelt H, Ziegler M, Perdas E (2018). Assessment and pathophysiology of pain in cardiac surgery. J Pain Res.

[17] Kang L, Zhang S-Y, Zhu W-L, Pang H-Y, Zhang L, Zhu M-L (2015). Is frailty associated with short-term outcomes for elderly patients with acute coronary syndrome?. J Geriatr Cardiol: JGC.

[18] Van Hecke O, Hocking LJ, Torrance N, Campbell A, Padmanabhan S, Porteous DJ (2017). Chronic pain, depression and cardiovascular disease linked through a shared genetic predisposition: analysis of a family-based cohort and twin study. PLoS ONE.

[19] Wang H, Zhao T, Wei X, Lu H, Lin X (2019). The prevalence of 30-day readmission after acute myocardial infarction: a systematic review and meta-analysis. Clin Cardiol.

[20] Kwok CS, Shah B, Al-Suwaidi J, Fischman DL, Holmvang L, Alraies C (2019). Timing and causes of unplanned readmissions after percutaneous coronary intervention: insights from the Nationwide Readmission Database. JACC Cardiovasc Interv.

[21] Ruddox V, Mathisen M, Otterstad JE (2012). Prevalence and prognosis of non-specific chest pain among patients hospitalized for suspected acute coronary syndrome-a systematic literature search. BMC Med.

[22] Spalding L, Reay E, Kelly C (2003). Cause and outcome of atypical chest pain in patients admitted to hospital. J R Soc Med.

[23] McManus DD, Saczynski JS, Lessard D, Waring ME, Allison J, Parish DC (2016). Reliability of predicting early hospital readmission after discharge for an acute coronary syndrome using claims-based data. Am J Cardiol.

[24] Crede SH, O'Keeffe C, Mason S, Sutton A, Howe E, Croft SJ (2017). What is the evidence for the management of patients along the pathway from the emergency department to acute admission to reduce unplanned attendance and admission? An evidence synthesis. BMC Health Serv Res.

[25] O'Cathain A, Knowles E, Maheswaran R, Pearson T, Turner J, Hirst E (2014). A system-wide approach to explaining variation in potentially avoidable emergency admissions: national ecological study. BMJ Qual Saf.

[26] Ricketts TC, Randolph R, Howard HA, Pathman D, Carey T (2001). Hospitalization rates as indicators of access to primary care. Health Place.

[27] Ware JE, Gandek B (1998). Overview of the SF-36 Health Survey and the International Quality of Life Assessment (IQOLA) Project. J Clin Epidemiol.

[28] Spertus JA, Winder JA, Dewhurst TA, Deyo RA, Prodzinski J, McDonnell M (1995). Development and evaluation of the Seattle Angina Questionnaire: a new functional status measure for coronary artery disease. J Am Coll Cardiol.

[29] Koleck TA, Dreisbach C, Bourne PE, Bakken S (2019). Natural language processing of symptoms documented in free-text narratives of electronic health records: a systematic review. J Am Med Inform Assoc.

[30] Pakhomov SV, Jacobsen SJ, Chute CG, Roger VL (2008). Agreement between patient-reported symptoms and their documentation in the medical record. Am J Manag Care.

[31] Heintzelman NH, Taylor RJ, Simonsen L, Lustig R, Anderko D, Haythornthwaite JA (2013). Longitudinal analysis of pain in patients with metastatic prostate cancer using natural language processing of medical record text. J Am Med Inform Assoc.

[32] Elkin PL, Froehling DA, Wahner-Roedler DL, Brown SH, Bailey KR (2012). Comparison of natural language processing biosurveillance methods for identifying influenza from encounter notes. Ann Intern Med.

[33] Nunes AP, Loughlin AM, Qiao Q, Ezzy SM, Yochum L, Clifford CR (2017). Tolerability and effectiveness of exenatide once weekly relative to basal insulin among type 2 diabetes patients of different races in routine care. Diabetes Ther.

[34] Tamang S, Patel MI, Blayney DW, Kuznetsov J, Finlayson SG, Vetteth Y (2015). Detecting unplanned care from clinician notes in electronic health records. J Oncol Pract.

[35] Chen J, Gagnier M, Lessard D, Kiefe C, McManus D, Wang B, et al. Validating natural language processing for extracting pain symptoms from electronic health records. In: The 2020 American Medical Informatics Association (AMIA) virtual annual symposium (poster); Nov 14–18, 2020.

[36] Savova GK, Masanz JJ, Ogren PV, Zheng J, Sohn S, Kipper-Schuler KC (2010). Mayo clinical Text Analysis and Knowledge Extraction System (cTAKES): architecture, component evaluation and applications. J Am Med Inform Assoc.

[37] Cuzick J (1985). A Wilcoxon-type test for trend. Stat Med.

[38] StataCorp,  (2017). Stata statistical software: release 15.

[39] Goldberg RJ, Saczynski JS, McManus DD, Waring ME, McManus R, Allison J (2015). Characteristics of contemporary patients discharged from the hospital after an acute coronary syndrome. Am J Med.

[40] Ritchie CS, Houston TK, Richman JS, Sobko HJ, Berner ES, Taylor BB (2016). The E-Coach technology-assisted care transition system: a pragmatic randomized trial. Transl Behav Med.

[41] Forman DE, Maurer MS, Boyd C, Brindis R, Salive ME, Horne FM (2018). Multimorbidity in older adults with cardiovascular disease. J Am Coll Cardiol.

[42] Singh S, Richter JE, Hewson EG, Sinclair JW, Hackshaw BT (1992). The contribution of gastroesophageal reflux to chest pain in patients with coronary artery disease. Ann Intern Med.

[43] Leise MD, Locke III GR, Dierkhising RA, Zinsmeister AR, Reeder GS, Talley NJ. Patients dismissed from the hospital with a diagnosis of noncardiac chest pain: cardiac outcomes and health care utilization. Mayo Clin Proc. 2010;85(4):323–330. 10.4065/mcp.2009.0428. 10.4065/mcp.2009.0428 PMC2848420 20194143

[44] Fass R, Achem SR (2011). Noncardiac chest pain: epidemiology, natural course and pathogenesis. J Neurogastroenterol Motil.

[45] Frieling T (2018). Non-cardiac chest pain. Visceral Med.

[46] Hadlandsmyth K, Rosenbaum DL, Craft JM, Gervino EV, White KS (2013). Health care utilisation in patients with non-cardiac chest pain: a longitudinal analysis of chest pain, anxiety and interoceptive fear. Psychol Health.

[47] Bhuiya FA. Emergency department visits for chest pain and abdominal pain: United States, 1999–2008: US Department of Health and Human Services, Centers for Disease Control and and Prevention, National Center for Health Statistics. 2010.

[48] Campbell KA, Madva EN, Villegas AC, Beale EE, Beach SR, Wasfy JH (2017). Non-cardiac chest pain: a review for the consultation-liaison psychiatrist. Psychosomatics.

[49] Centers for Medicare & Medicaid Services. Medicare Program: CY 2020 revisions to payment policies under the physician fee schedule and other changes to part B payment policies. Medicare Shared Savings Program Requirements Final Rule. 2019; 84(221). https://www.federalregister.gov/documents/2019/11/15/2019-24086/medicare-program-cy-2020-revisions-to-payment-policies-under-the-physician-fee-schedule-and-other. Accessed 20 Oct 2020.

[50] Naylor MD, Brooten D, Campbell R, Jacobsen BS, Mezey MD, Pauly MV (1999). Comprehensive discharge planning and home follow-up of hospitalized elders: a randomized clinical trial. JAMA.

[51] Jack BW, Chetty VK, Anthony D, Greenwald JL, Sanchez GM, Johnson AE (2009). A reengineered hospital discharge program to decrease rehospitalization: a randomized trial. Ann Intern Med.

[52] Coleman EA, Parry C, Chalmers S, Min SJ (2006). The care transitions intervention: results of a randomized controlled trial. Arch Intern Med.

