# Supplementary material for: Non-specific pain and 30-day readmission in acute coronary syndromes: findings from the TRACE-CORE prospective cohort
Source: BMC Cardiovasc Disord. 2021 Aug 9;21:383. doi: 10.1186/s12872-021-02195-z (PMC8351351; doi:10.1186/s12872-021-02195-z)
Supplement: Supplementary file 1 — Additional file 1. Comparison of characteristics between patients analyzed in this study and all TRACE-CORE participants. [file 12872_2021_2195_MOESM1_ESM.pdf]

# Supplement 1. Comparison of characteristics of patients analyzed in this study and all TRACE-CORE participants

### Table S1-1. Patient characteristics for this study (N=787) and the full TRACE-CORE population (N=2174): TRACE-CORE, 2011-2013

| Characteristic | This Study (N = 787) | Full TRACE-CORE population (N = 2174) |
|---|---|---|
| Age (mean, y) | 62.0 | 61.3 |
| Female (%) | 29.9 | 33.5 |
| Non-Hispanic white (%) | 94.9 | 81.0 |
| Education | | |
| ≤High school | 36.3 | 46.5 |
| Some college | 30.6 | 28.9 |
| ≥College graduate | 33.0 | 24.7 |
| Live Alone | 20.1 | 21.9 |
| Low Health Literacy | 29.0 | 36.1 |
| Heavy Drinker | 9.4 | 9.6 |
| Current Smoker | 20.6 | 23.3 |

The statistics for patient characteristics for all the TRACE-CORE participants were calculated by using information from a published TRACE-CORE paper.[1]

References:

1. Goldberg RJ, Saczynski JS, McManus DD, et al. Characteristics of contemporary patients discharged from the hospital after an acute coronary syndrome. *The American journal of medicine.* 2015;128(10):1087-1093.
