# Supplementary material for: Non-specific pain and 30-day readmission in acute coronary syndromes: findings from the TRACE-CORE prospective cohort
Source: BMC Cardiovasc Disord. 2021 Aug 9;21:383. doi: 10.1186/s12872-021-02195-z (PMC8351351; doi:10.1186/s12872-021-02195-z)
Supplement: Supplementary file 2 — Additional file 2. Example cases where NLP identified pain not reported by the SF-36 survey. [file 12872_2021_2195_MOESM2_ESM.pdf]

Supplement 2. Example cases where NLP identified pain not reported by the SF-36 survey

Category	Number of cases, N (%)	Examples
Total	35 (100)	
NLP is correct	20 (59%)	"He did ask that for headaches it would be okay if he use Tylenol, which I said was fine."
		I obtained a 12 lead EKG on #patient# based on his episode of pain at home for which he took a nitroglycerin.
		polymyalgia rheumatica. We will slowly taper his prednisone. He had gotten down to 6 mg without any trouble, but when he dropped to 5, his symptoms returned. As mentioned previously, I will decrease to 10 mg daily for two weeks and then 9 mg daily for two weeks before following up with me.
NLP is wrong	9 (26%)	
History	2	"ASSESSMENT AND PLAN. In summary, this is an … The patient was hospitalized for chest pain."
Negation	5	"He has done well since his procedure without symptoms of angina or shortness of breath."
		"His pain is good."
Hypothetical	1	"He was warned about the potential side effects including but not limited to lactic acidosis which would

		be experiencing the stomach pains, nausea, vomiting and muscle aches, if he experiences any of these things, he should stop it immediately and call the clinic. …”
Typos in EHR	1	“ASSESSMENT AND PLAN … Status post cardiac catheterization, which believes to have an 80% lesion to the left circ with a drug eluting stent. Other vessels previously sent [**stent**] did appear to be pain [**patent**] and as per the report.”
Ambiguous cases	5 (15%)	“MEDICATIONS … Nitrostat 0.4 mg every 5 minutes x3 p.r.n. pain”

Patient-self-reported pain status was collected for the first month after discharge from index hospitalization (using SF-36 survey distributed at one month post-discharge). Electronic health record (EHR) documentation of pain was extracted by natural language processing from patient's clinical notes created within 30 days post discharge and before readmission to the hospital (if any).

NLP natural language processing; *EHR* electronic health record.
Among the 9 erroneous cases, 5 (50%) were negations of pain, 2 (20%) documented chest pain as history but not in the section “History of Present Illness”, 1 (10%) was hypothetical pain, and 1 (10%) was caused by a typo in the EHR note. NLP-identified pain symptoms were underlined.
