# Supplementary material for: Non-specific pain and 30-day readmission in acute coronary syndromes: findings from the TRACE-CORE prospective cohort
Source: BMC Cardiovasc Disord. 2021 Aug 9;21:383. doi: 10.1186/s12872-021-02195-z (PMC8351351; doi:10.1186/s12872-021-02195-z)
Supplement: Supplementary file 3 — Additional file 3. Sensitivity analyses, using patients not self-reporting chest pain (N = 530). [file 12872_2021_2195_MOESM3_ESM.pdf]

Supplement 3. Sensitivity analyses, using patients not self-reporting chest pain (N=530)

Table S3-1. Patient characteristics at baseline, by pain self-reported one month after discharge: TRACE-CORE, 2011-2013

**Variable, n (%) or mean [SD]**	**All Patients**	**Categories of Self-Reported Pain after Discharge**				
		**No pain**	**Mild-moderate pain, documented in EHR**	**Mild-moderate pain, NOT documented in EHR**	**Severe pain**	***P* value[1]**
	**N = 530**	**N = 255**	**N = 60**	**N =191**	**N = 24**	
	N (%)	N (%)	N (%)	N (%)	N (%)	
Age						0.24
<50	70 (13.2)	28 (11.0)	6 (10.0)	31 (16.2)	5 (20.8)	
50-64	218 (41.1)	108 (42.4)	31 (51.7)	69 (36.1)	10 (41.7)	
≥65	242 (45.7)	119 (46.7)	23 (38.3)	91 (47.6)	9 (37.5)	
Gender						0.08
Male	386 (72.8)	194 (76.1)	48 (80.0)	128 (67.0)	16 (66.7)	
Female	144 (27.2)	61 (23.9)	12 (20.0)	63 (33.0)	8 (33.3)	
Race						0.43
White	503 (95.8)	242 (95.7)	59 (100.0)	180 (95.2)	22 (91.7)	
Black	4 (0.8)	3 (1.2)	0 (0.0)	1 (0.5)	0 (0.0)	
Hispanic	18 (3.4)	8 (3.2)	0 (0.0)	8 (4.2)	2 (8.3)	
Education						0.45
≤High school	191 (36.0)	100 (39.2)	18 (30.0)	62 (32.5)	11 (45.8)	
Some college	157 (29.6)	69 (27.1)	17 (28.3)	64 (33.5)	7 (29.2)	
≥College graduate	182 (34.3)	86 (33.7)	25 (41.7)	65 (34.0)	6 (25.0)	
Low Social Support						0.99
No	501 (94.5)	240 (94.1)	57(95.0)	181 (94.8)	23 (95.8)	
Yes	29 (5.5)	15 (5.9)	3 (5.0)	10 (5.2)	1 (4.2)	
Live Alone						0.86
No	427 (80.6)	206 (80.8)	46 (76.7)	156 (81.7)	19 (79.2)	
Yes	103 (19.4)	49 (19.2)	14 (23.3)	35 (18.3)	5 (20.8)	
Low Health Literacy						0.71
No	379 (71.9)	182 (71.7)	39 (66.1)	140 (73.7)	18 (75.0)	
Yes	148 (28.1)	72 (28.3)	20 (33.9)	50 (26.3)	6 (25.0)	
Heavy Drinker						0.88
No	441 (90.9)	233 (91.4)	53 (88.3)	174 (91.1)	22 (91.7)	

Yes	44 (9.1)	22 (8.6)	7 (11.7)	17 (8.9)	2 (8.3)	
Current Smoker						0.03*
No	432 (81.5)	210 (82.4)	51 (85.0)	157 (82.2)	14 (58.3)	
Yes	98 (18.5)	45 (17.6)	9 (15.0)	34 (17.8)	10 (41.7)	
Where to seek health care						0.15
No places	56 (10.6)	25 (9.9)	7 (11.7)	21 (11.1)	3 (12.5)	
Clinic or doctor office	426 (81.0)	209 (82.9)	47 (78.3)	153 (80.5)	17 (70.8)	
Other places	24 (4.6)	8 (3.2)	2 (3.3)	13 (6.8)	1 (4.2)	
Emergency department	20 (3.8)	10 (4.0)	4 (6.7)	3 (1.6)	3 (12.5)	
ACS type						0.005*
STEMI	123 (23.2)	65 (25.5)	5 (8.3)	46 (24.1)	7 (29.2)	
NSTEMI	314 (59.2)	156 (61.2)	37 (61.7)	106 (55.5)	15 (62.5)	
Unstable angina	93 (17.5)	34 (13.3)	18 (30.0)	39 (20.4)	2 (8.3)	
History of PCI						0.37
No	422 (79.6)	208 (81.6)	47 (78.3)	161 (79.1)	16 (66.7)	
Yes	108 (20.4)	47 (18.4)	13 (21.7)	40 (20.9)	8 (33.3)	
History of CABG						0.64
No	481 (90.8)	231 (90.6)	52 (86.7)	176 (92.1)	22 (91.7)	
Yes	49 (9.2)	24 (9.4)	8 (13.3)	15 (7.9)	2 (8.3)	
Comorbidity [a] (mean [SD])	1.9 [1.6]	1.8 [1.6]	2.2 [1.8]	1.9 [1.5]	2.5 [1.5]	0.06
Reporting generic pain						<0.001*
No	145 (27.6)	94 (37.2)	7 (11.7)	40 (21.2)	4 (16.7)	
mild to moderate	299 (56.8)	129 (51.0)	41 (68.3)	119 (63.0)	10 (41.7)	
severe	82 (15.6)	30 (11.9)	12 (20.0)	30 (15.9)	10 (41.7)	

Pain category was defined using pain self-reported for the first month after discharge from index hospitalization (using SF-36 survey distributed at one month post-discharge) and electronic health record (EHR) documentation of pain extracted by natural language processing from patient's clinical notes created within 30 days post discharge and before readmission to the hospital (if any). *TRACE-CORE* Transitions, Risks, and Actions in Coronary Events Center for Outcomes Research and Education, *STEMI* ST-segment elevation myocardial infarction, *NSTEMI* non-ST segment elevation myocardial infarction, *PCI* percutaneous coronary intervention, *CABG* coronary artery bypass graft, *SD*, standard deviation

[a] The number of diseases from the following 14 conditions in patient's medical history: atrial fibrillation, Alzheimer or dementia, anemia, cancer, congestive heart failure, chronic kidney disease, chronic lung disease, Type 2 diabetes, dialysis, hypertension, peripheral vascular disease, valvular heart disease, coronary heart disease or myocardial infarction, transient ischemic attack or stroke.

[*] indicates statistically significant ($P<0.05$). *p*-values were calculated by chi-square test (for categorical variables that have no cell size smaller than 5), Fisher's exact test (for categorical variables that have cell size smaller than 5), and analysis of variance (for comorbidity).

Table S3-2. Association of self-reported pain status at one month with 30-day rehospitalization: TRACE-CORE, 2011-2013

**Pain condition**	Incidence of 30-day rehospitalization	Model 1[a]: Unadjusted		Model 2[b]: Adjusted for baseline characteristics	
	n/N (%)	OR (95% CI)	*P* value	OR (95% CI)	*P* value
**General pain by severity**					
Reporting no pain	17/255 (6.7)	Reference		Reference	
Reporting mild to moderate pain	23/251 (9.2)	1.41 (0.74-2.71)	0.3	1.43 (0.73-2.82)	0.30
Reporting severe pain	4/24 (16.7)	2.80 (0.86-9.12)	0.09	2.55 (0.73-8.93)	0.14

Patient-self-reported pain status was collected for the first month after discharge from index hospitalization (using SF-36 survey distributed at one month post-discharge).

TRACE-CORE Transitions, Risks, and Actions in Coronary Events Center for Outcomes Research and Education

[a] Model 1: logistic regression, unadjusted for covariates.

[b] Model 2: multivariable logistic regression, adjusted for generic pain, smoking status, acute coronary syndrome type, and healthcare seeking behaviors reported at index hospitalization.

[*] indicates statistically significant ($P<0.05$).

Table S3-3. Association of pain category (combining self-report with EHR data) with 30-day rehospitalization: TRACE-CORE, 2011-2013

**Pain condition**	Incidence of 30-day rehospitalization	Model 1[a]: Unadjusted		Model 2[b]: Adjusted for baseline characteristics	
	n/N (%)	OR (95% CI)	*P* value	OR (95% CI)	*P* value
**Generic pain**					
Reporting no pain	17/255 (6.7)	Reference		Reference	
Reporting mild to moderate pain, documented in EHR	5/60 (8.3)	1.27 (0.45-3.60)	0.65	1.27 (0.43-3.74)	0.67
Reporting mild to moderate pain, not documented in EHR	18/191 (9.4)	1.46 (0.73-2.91)	0.29	1.48 (0.73-3.02)	0.28
Reporting severe pain	4/24 (16.7)	2.8 (0.86-9.12)	0.09	2.54 (0.73-8.90)	0.14

Patient-self-reported pain status was collected for the first month after discharge from index hospitalization (using SF-36 survey distributed at one month post-discharge). Electronic health record (EHR) documentation of pain was extracted by natural language processing from patient's clinical notes created within 30 days post discharge and before readmission to the hospital (if any).

TRACE-CORE indicates Transitions, Risks, and Actions in Coronary Events Center for Outcomes Research and Education

[a] Model 1: logistic regression, unadjusted for covariates.

[b] Model 2: multivariable logistic regression, adjusted for generic pain, smoking status, acute coronary syndrome type, and healthcare seeking behaviors reported at index hospitalization.

[*] indicates statistically significant ($P<0.05$).
